# Unusual Initial Presentation of Herpes Simplex Virus as Inguinal Lymphadenopathy

**DOI:** 10.1155/2015/573230

**Published:** 2015-02-26

**Authors:** Sarah A. Fleming, John G. Strickler

**Affiliations:** Hospital Pathology Associates, 2800 10th Avenue South Suite 2200, Minneapolis, MN 55407, USA

## Abstract

Genital herpes simplex virus (HSV) infections are a common cause of inguinal lymphadenopathy. However, surgical excision of enlarged inguinal nodes is almost never performed to initially diagnose genital herpes simplex virus, due to the distinct external presentation of genital herpetic vesicles that usually occur with the first symptoms of infection. Therefore, the histologic and immunophenotypic features of HSV-associated inguinal lymphadenopathy are unfamiliar to most pathologists. The current report describes the lymph node pathology of two immunocompetent patients, whose initial HSV diagnosis was established through surgical excision of enlarged inguinal lymph nodes. Histologic examination showed features consistent with viral lymphadenopathy, including florid follicular hyperplasia, monocytoid B-cell hyperplasia, and paracortical hyperplasia without extensive necrosis. Immunohistochemical stains for HSV antigens, using polyclonal anti-HSV I and II antibodies, demonstrate strong immunoreactivity for HSV in a small number of cells in the subcapsular sinuses, especially in areas with monocytoid B-cell hyperplasia. Rare scattered HSV-positive cells also are identified in paracortical areas and germinal centers. We conclude that an initial diagnosis of genital HSV infection may be established by inguinal lymph node biopsy.

## 1. Introduction

Surgical excision of enlarged inguinal lymph nodes is often performed to exclude hematologic malignancies. These lymph node biopsy specimens often show lymphoid hyperplasia, which may be due to a wide variety of reactive conditions including various sexually transmitted infections [1]. However, it is unusual for genital herpes simplex virus (HSV) to present initially as inguinal lymphadenopathy, which is significant enough to warrant nodal excision, unless the patient also has a known underlying lymphoma [2]. Although the initial histologic diagnosis of genital HSV by inguinal lymph node biopsy is rare [3], we now describe two immunocompetent patients with active genital herpes diagnosed by inguinal lymph node biopsy.

## 2. Materials and Methods

Formalin-fixed, paraffin-embedded tissue and hematoxylin and eosin-stained slides were studied in both cases. Immunohistochemical studies on paraffin-embedded material were performed, using antibodies for herpes simplex virus (HSV) I and II following fixation in 10% neutral buffered formalin. The rabbit polyclonal HSV I and II antibodies from Cell Marque (CMC36111031, CMC36211031, Rocklin, California) were used at a dilution of 1 : 200 for HSV I and 1 : 50 for HSV 2. Immunostaining was performed using the Leica Bond-Max automation system and Leica Refine detection kit (Leica Biosystems, Bannockburn, IL). The approximately 3-hour protocol included online deparaffinization and low-pH epitope retrieval for 10 minutes, incubation with primary antibodies for 15 minutes, postprimary IgG linker reagent (rabbit anti-mouse IgG) for 8 minutes, polymer anti-rabbit HRP IgG for 8 minutes, and DAB as the chromogen for 10 minutes, followed by 5-minute hematoxylin counterstaining. A positive control was run with each sample.

## 3. Case Presentation

**Figure 1:**
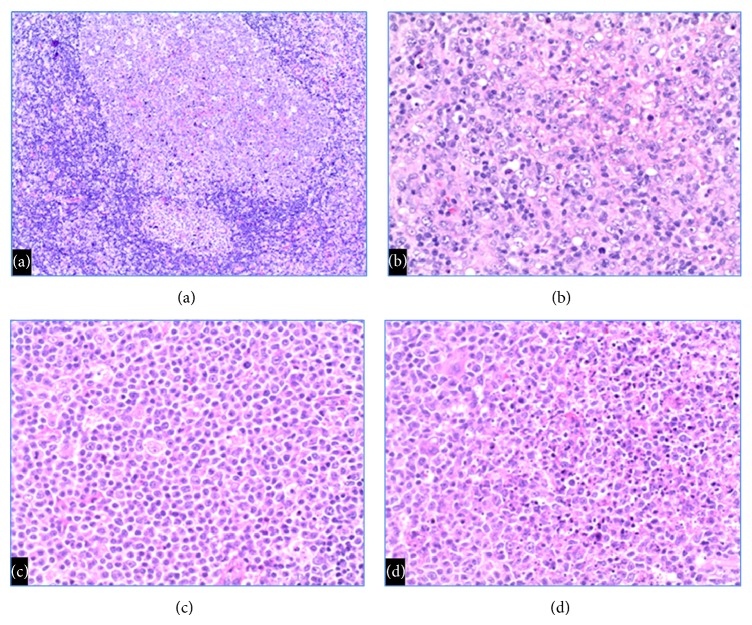
Histologic sections show (a) follicular hyperplasia with attenuated mantle zones and adjacent monocytoid B-cell hyperplasia (Case 1) 100x magnification, (b) paracortical hyperplasia with a moderate number of immunoblasts (Case 1) 200x magnification, (c) monocytoid B-cell hyperplasia (Case 1) 200x magnification, and (d) monocytoid B-cell hyperplasia with focal necrosis (Case 2) 200x magnification.

Both patients presented clinically with enlarged and tender inguinal lymph nodes. CT scans confirmed enlarged inguinal nodes (both measuring greater than 3 cm in diameter), without evidence of systemic lymphadenopathy. Lymph node biopsies in both patients were performed to exclude lymphoma and other malignancies. Biopsy specimens from the inguinal lymph nodes showed similar histologic features (Figure 1).

### 3.1. Case 1

The patient is a 16-year-old female, who had a 3.7 × 3.5 × 1.7 cm inguinal lymph node removed. Histologic examination showed florid follicular hyperplasia including some follicles with attenuated mantle zones. Subsequent HIV serology studies were negative. Monocytoid B-cell hyperplasia was prominent. Extranodal marginal zone lymphoma, which occasionally is difficult to distinguish from monocytoid B-cell hyperplasia, was excluded by absence of diffuse architectural effacement and by absence of a monoclonal B-cell population by flow cytometry. The lymph node also showed paracortical hyperplasia, including a moderate number of immunoblasts with prominent nucleoli, but absence of Reed-Sternberg cells/variants excluded Hodgkin lymphoma.

### 3.2. Case 2

The patient is a 43-year-old male who had a 3 × 2 × 1 cm inguinal lymph node removed. Histologic examination shows florid follicular hyperplasia with minimal paracortical hyperplasia. Some of the sinusoidal areas show monocytoid B-cell hyperplasia, focally associated with necrosis. Histologic examination revealed no evidence of hematologic malignancy, and flow cytometry confirmed the absence of lymphoma.

**Figure 2:**
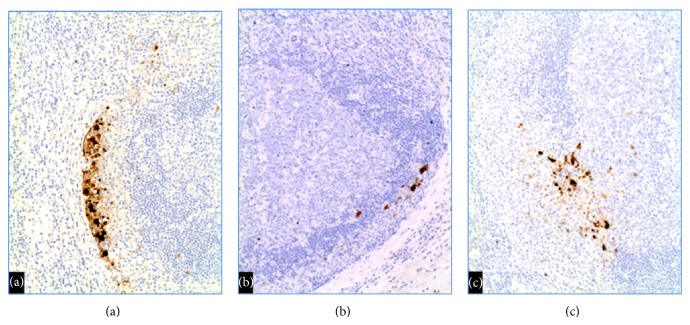
Immunohistochemical stains show small numbers of HSV-I/HSV-II positive cells in (a) subcapsular sinus (Case 2) 40x magnification, (b) subcapsular sinus (Case 1) 100x magnification, and (c) areas of monocytoid B-cell hyperplasia (Case 2) 100x magnification.

Immunohistochemical stains (Figure 2) of both cases show small numbers of HSV-I/HSV-II positive cells in the lymph node sinuses, predominantly in the subcapsular sinus. The viral inclusions were found mainly in sinuses containing monocytoid B-cell hyperplasia, with rare scattered HSV-positive cells in the paracortex, and rare weak HSV-positive cells in germinal centers. The HSV-infected cells appear to be mainly macrophages (CD68-positive) rather than B-lymphocytes (CD20-positive). The significance of these findings in the HSV-associated immune response is not clear.

Immunostains for CMV, cat scratch disease, and toxoplasmosis were negative. A small number of EBV positive lymphoid cells were present in Case 1, indicative of a prior EBV infection rather than acute EBV associated infectious mononucleosis.

## 4. Discussion

An initial diagnosis of HSV-associated lymphadenopathy was established by inguinal lymph node biopsy in both of our patients. The patients were immunocompetent, presented with palpable tender inguinal nodes, and did not have current or prior genital herpes infection or hematologic malignancy. Localized lymphadenopathy has been reported in up to 80% of patients who have an active genital HSV infection [4]. However, lymph node biopsy is very rarely performed for initial diagnostic purposes, as genital HSV infections usually present with predominant local symptoms such as pain, itching, dysuria, vaginal or urethral discharge, or lesions before tender lymphadenopathy develops [4].

Our patients were both young sexually active individuals, suggesting that both had recent exposure to HSV, which presented initially as inguinal lymphadenopathy. In an autopsy review study of disseminated herpes disease, HSV was never found as an incidental finding in lymphoid tissues, except in patients with an underlying hematologic malignancy [5]. In addition, previous studies have shown a correlation between HSV derived inguinal lymphadenopathy and subsequent development of genital HSV-related lesions [6, 7].

**Figure 3:**
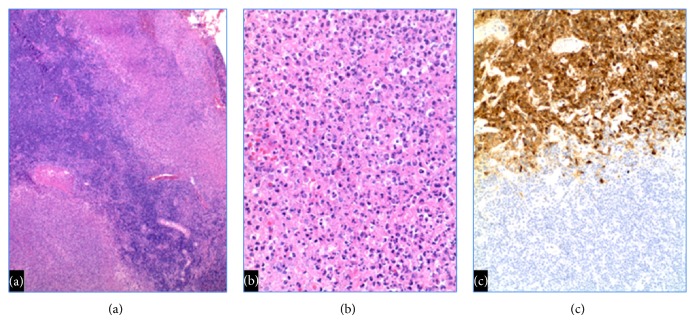
HSV infections when superimposed on low grade B-cell lymphomas show (a) well-demarcated areas of necrosis (Reference case) 40x magnification, (b) necrosis containing HSV viral inclusions (Reference case) 200x magnification, and (c) numerous viral inclusions only in the necrotic areas by HSV immunohistochemistry (Reference case) 100x magnification.

Previous reports of biopsy-proven HSV-associated lymphadenopathy indicate that many patients are immunocompromised and/or have underlying low-grade B-cell lymphomas [6, 8, 9]. Neither of our patients showed histologic or immunologic evidence of concurrent lymphoma. Simultaneous nodal involvement by HSV and low-grade B-cell lymphoma (Figure 3) is characterized by diffuse effacement of nodal architecture, with well-demarcated foci of necrosis containing numerous HSV-I/HSV-II infected cells.

The histologic appearance in our two patients (florid follicular hyperplasia, monocytoid B-cell hyperplasia, and paracortical hyperplasia) overlaps with lymphadenopathy associated with other viral-associated lymphadenopathy, and HSV immunohistochemistry is essential for establishing the correct diagnosis. Note that monocytoid B-cell hyperplasia may be related to memory B-cell reactivation, although this histologic pattern also is commonly associated with acute viral lymphadenopathy. Cytomegalovirus (CMV) lymphadenopathy has similar histologic features, including prominent follicular hyperplasia and monocytoid B-cell hyperplasia. Cytomegalovirus inclusions (typically within the monocytoid B-cell hyperplasia) can be identified histologically and confirmed by immunohistochemistry. The lymphadenopathy associated with acute EBV-associated infectious mononucleosis has more prominent paracortical hyperplasia than HSV-associated lymphadenopathy. EBV in situ hybridization studies (on tissue sections) or serologic studies are required for confirmation. The follicular hyperplasia associated with human immunodeficiency virus (HIV) infections is typically characterized by absent mantle zones and follicle lysis. The mantle zones in our first patient were somewhat attenuated, but HIV serologic studies excluded concurrent HIV-associated lymphadenopathy.

Although rare, HSV can initially present as inguinal lymphadenopathy without other associated symptoms. We conclude that HSV-associated lymphadenopathy should be considered whenever enlarged inguinal lymph nodes show a viral histologic appearance, especially when encountered in young, sexually active adults.
